# Modern Treatment of Diphtheria in Private Practice

**Published:** 1896-12

**Authors:** 


### Abstracts and Selections.

### The Modern Treatment of Diphtheria in Private Practice.

Dr. W. A. Walker, of New York, in Pediatrics for October, 1896, says:

The results of treatment with anti-diphtheritic serum, stands in bold contrast with the drug treatment with the bottles of medicine, the cruel swab, the sleepless nights, the futile attemp to force food and medicine, the onset of secondary infection, and death or a tardy convalescence.

The uniform success which I have observed, and had in my own practice, has convinced me that the treatment of diphtheria with antitoxin is a great advance in therapeutics, and it is my impression that critics who have condemned this treatment have in most instances either observed only hospital patients, or have not persisted in the treatment, or perhaps have not had a fresh and reliable serum, or have not used it early enough.

From the standpoint of a general practitioner, I confidently expect to cure any case of diphtheria in private practice seen with in forty-eight hours of the onset of the disease.

Take, for instance, a typical case: a previously healthy child six years of age. The family physician is called in and finds the following conditions: general depression, face pale, pulse accelerated, temperature about 101° F. Inspection of throat shows general diffuse redness, with the characteristic deposit on one or both tonsils. This peculiar deposit once seen is not readily forgotten; the high fever, flushed face, and rapid pulse usually seen in pseudo-membranous tonsillitis are absent; the margin of the inflammatory process is usually sharply defined in diphtheria and not in tonsillitis. In follicular tonsillitis the leading symptoms are: intense congestion of the tonsils, with small discrete white patches, pulse and temperature high.

If, however, the symptoms are not well defined, and the differential diagnosis cannot be clearly made, we should give the patient the benefit of the doubt and a dose of anti-diphtheritic serum administered at once. Then a culture should be made to verify the diagnosis. If I believe the case to be diphtheria, or have a reasonable doubt as to the diagnosis, I use the antitoxin whilst waiting for the report from the bacteriologist. If the case turns out to be tonsillitis, no harm has been done, as I consider a fresh, reliable serum, properly administered, devoid of danger.

Given, then, a case where the diagnosis of diphtheria is clear, I give as quickly as possible, either 1000 units or 1500 units of the serum. The attendant is instructed to keep the throat clean with a bichloride solution of 1 to 5000; or a solution of permanganate of potash may be used, 1 to 4000, if the attendant is not a trained nurse. With a young child, difficult to manage, it is best to inject the solution into the nostrils; in older children a spray can be used in both the nostrils and throat more advantageously.

At the end of twenty-four hours I expect to find the membrane beginning to shrivel and curl up at the edges. In any event, however, I administer a second injection at this stage of the disease, and in a majority of instances this is sufficient. I advise very strongly that the second injection be given in all cases where the diagnosis of diphtheria is clear. I do not expect a cure from one injection, and rarely omit the second. If the symptoms do not indicate the beginning of convalescence at the end of forty-eight hours, I give a third injection. In fact, I would use a fourth injection if it seemed advisable at the end of another twenty-four hours, but I think this will rarely be found necessary.

I have not used anti-streptococci serum, but I am convinced that in cases in which the treatment has been delayed, or in cases showing the streptococcic infection, proven by bacteriological investigation, or from the peculiar red zone of inflammation which begins to spread from the margin of the diphtheritic process, the anti-streptococcic serum should promptly be used. Not only would I do this, but in case of severe acute disease in the throat, which present all the symptoms of diphtheria, but where the bacteriologist's report does not confirm the diagnosis, I would resort to the anti-streptococcic serum. In fact, if I should have a case of diphtheria in which the membrane does not begin to peel up by the end of the twenty-four hours following, say, the second injection of anti-toxin, I will use the anti-streptococcic serum.

The importance of a fresh, reliable, highly concentrated serum must not be lost sight of, and as I have full confidence in our American products, I do not use imported serums. I have used several serums, but have been best satisfied with the effects of that sent out from the biological department of Parke, Davis & Co. I heartily approve of the way this firm now puts up the serum, in bulbs instead of bottles. It is not only highly concentrated, but, being hermetically sealed, should keep indefinitely. It is put up in bulbs of so many units, 250, 500, 1000, 1500, and each bulb being a dose, there is no temptation to use a serum that has been exposed to the atmosphere.

As to the medicinal treatment, I do not give any drug with the idea of influencing the course of the disease. I treat the conditions as they arise, symptomatically. If I have evidence of the absorption of poisonous secretions, and a coated tongue, I give calomel tablet triturates, ¼ grain every hour, until the bowels move freely. Alcohol is rarely needed in cases receiving the serum treatment, especially if it is used early enough, whereas, under the old treatment, when we were so apt to find profound toxic symptoms, alcohol was more often needed. It is perhaps well to state here that I prefer fluid nourishment, principally milk, during the course of the disease.

---

**Holiday Excursions.**—The I. & G. N. R. R. will, as usual, sell holiday excursion tickets to points in the Southeast, December 21st and 22nd at one fare rates, limited thirty days from date of sale for return.

Local excursions will also be run for Christmas and New Years. Call on ticket agent of I. & G. N. R. R. for further information or address, D. J. PRICE, A. G. P. A.

